# Predictive Model for the Risk of Severe Acute Malnutrition in Children

**DOI:** 10.1155/2019/4740825

**Published:** 2019-07-01

**Authors:** Olivier Mukuku, Augustin Mulangu Mutombo, Lewis Kipili Kamona, Toni Kasole Lubala, Paul Makan Mawaw, Michel Ntetani Aloni, Stanislas Okitotsho Wembonyama, Oscar Numbi Luboya

**Affiliations:** ^1^Department of Research, Institut Supérieur des Techniques Médicales, Lubumbashi, Democratic Republic of the Congo; ^2^Department of Pediatrics, University Hospital of Lubumbashi, University of Lubumbashi, Lubumbashi, Democratic Republic of the Congo; ^3^School of Public Health, University of Lubumbashi, Lubumbashi, Democratic Republic of the Congo; ^4^Division of Hemato-oncology and Nephrology, Department of Pediatrics, University Hospital of Kinshasa, School of Medicine, University of Kinshasa, Kinshasa, Democratic Republic of the Congo

## Abstract

**Background:**

The nutritional status is the best indicator of the well-being of the child. Inadequate feeding practices are the main factors that affect physical growth and mental development. The aim of this study was to develop a predictive score of severe acute malnutrition (SAM) in children under 5 years of age.

**Methods:**

It was a case-control study. The case group (*n* = 263) consisted of children aged 6 to 59 months admitted to hospital for SAM that was defined by a *z*-score weight/height < −3 SD or presence of edema of malnutrition. We performed a univariate and multivariate analysis. Discrimination score was assessed using the ROC curve and the calibration of the score by Hosmer–Lemeshow test.

**Results:**

Low birth weight, history of recurrent or chronic diarrhea, daily meal's number less than 3, age of breastfeeding's cessation less than 6 months, age of introduction of complementary diets less than 6 months, maternal age below 25 years, parity less than 5, family history of malnutrition, and number of children under 5 over 2 were predictive factors of SAM. Presence of these nine criteria affects a certain number of points; a score <6 points defines children at low risk of SAM, a score between 6 and 8 points defines a moderate risk of SAM, and a score >8 points presents a high risk of SAM. The area under ROC curve of this score was 0.9685, its sensitivity was 93.5%, and its specificity was 93.1%.

**Conclusion:**

We propose a simple and efficient prediction model for the risk of occurrence of SAM in children under 5 years of age in developing countries. This predictive model of SAM would be a useful and simple clinical tool to identify people at risk, limit high rates of malnutrition, and reduce disease and child mortality registered in developing countries.

## 1. Background

Nutritional status is the best indicator of child well-being and indirectly the well-being of the community. In developing countries, feeding practices are very often inadequate and inconsistent with the World Health Organization (WHO) recommendations and are the main factors affecting the physical growth and mental development of the child [1]. Poor nutritional status in early childhood also affects health in adulthood [2]. The WHO estimated that severe acute malnutrition (SAM) affects about 20 million children under 5 years of age [3].

Although known to be a major public health problem in low-income countries, malnutrition contributes significantly to mortality among children under 5 years of age. In 2011, it was estimated that about 45% of deaths in children would be attributed to malnutrition [4, 5]. The Democratic Republic of Congo (DRC) is part of 5 countries in the world (India, Nigeria, Pakistan, and China) with a high mortality rate among children under 5 [6], and malnutrition is one of the leading causes of death in these countries, associated with other diseases such as diarrhea, pneumonia, and malaria, which are more frequent in children under 5 [5].

Infant malnutrition is influenced by multidimensional factors. According to Kikafunda, the factors that influence infant malnutrition in developing countries are divided into three groups: maternal factors, dietary and socioenvironmental factors, and economic factors [7]. A number of studies have shown that infant malnutrition is strongly entrenched in poverty [8–11]. However, the relationship between poverty and infant malnutrition is quite complex. Malnutrition affects poor households as well as nonpoor households [12, 13]. High household incomes cannot guarantee a satisfactory nutritional outcome for children if households lack hygienic care, food quality, and access to health care [14–16].

Developing countries in general, and the DRC in particular, face several challenges, such as deficient technical platform, underqualification of rural health staff, poor distribution or inadequate health services, and the difficulties of access to reference structures. Thus, based on these concerns, the WHO had defined the characteristics of an ideal screening test for resource-limited countries as being an affordable, sensitive, specific, user-friendly, and rapid test without equipment and delivered to those who need it most.

In this study, we focused on the sociodemographic and nutritional aspects that are readily available during preschool consultations or during immunization campaigns to enable us to predict the risk of SAM in children under 5 in our context.

No global study incorporating multivariate analysis has been published previously in the DRC and no published scores are suitable for predicting the risk of SAM in a population under 5 in developing countries. This study is the first in Lubumbashi to establish the independent character of the risk factors, which is important to deduce perspectives of action. This is the goal of this study, which is to develop a predictive score of SAM in children under 5.

## 2. Methods

### 2.1. Study Design

This is a case-control study conducted at the Jason Sendwe Hospital in Lubumbashi (DRC) that focused on children aged 6 to 59 months between January 1, 2011 and December 31, 2012. Case group consisted of children aged 6 to 59 months admitted to hospital for SAM that was defined by a *z*-score weight/height < −3 SD (calculated using WHO Anthro 2011 version 3.2.2) or children with edema of malnutrition according to WHO 2006 growth standards [17, 18]. The anthropometric and clinical parameters were recorded by the medical team working at the Intensive Nutritional Therapy Unit of this hospital, trained in anthropometry, assisted by the child's caretaker. Controls were recruited at each admission of a SAM case. The anthropometric and clinical parameters of these controls were recorded by the same team. For this, undressed or minimally dressed children were weighed using a SECA digital weighing machine and recumbent height was measured by using a height board. The management of SAM follows the steps of the WHO guidelines [19] adopted by the National Nutrition Program in DRC. The control group was composed of children of the same age who were seen in the same hospital for routine consultation or preschool consultation and who did not have severe diseases.

Our sampling was exhaustive; we recorded all cases of SAM admitted to the Jason Sendwe Hospital in this period. Cases and controls were included consecutively and prospectively, and the match was 1 by 1 depending on the date of consultation. The number of subjects included in the study was 263 cases and 263 controls. We excluded children with positive or unknown HIV status and those with a pathology that could influence growth or progression during hospitalization (spinal or lower limbs deformities, heart diseases, kidney diseases, chronic neuropathies, digestive abnormalities with malabsorption syndrome, and sickle cell disease).

The study was approved by the Medical Ethics Committee of the University of Lubumbashi. Patient record/information was anonymized and deidentified prior to analysis to ensure confidentiality of individual patient information.

### 2.2. Study Variables

The data were collected using a structured questionnaire via a face-to-face interview. The questionnaire was initially prepared in French, then translated into Swahili, and then translated back into French to check equivalence. The parents or guardians of individual study participants responded to the questionnaire.

Mothers were asked about the household in which the child had lived in the two months prior to admission, the history of breastfeeding, and the symptoms present. If the mother was present, she was asked to recall the birth weight of the child. For other children, we would refer to the child's growth monitoring booklet issued to the mother at the birth of the child. We studied the following variables:

1. Characteristics and personal history of the child: age, sex, low birth weight (defined by birth weight <2500 grams), recurrent/chronic diarrhea, and follow-up of preschool consultations. Recurrent/chronic diarrhea is defined as three soft or liquid stools for at least 24 hours, two or more weeks prior to the deterioration of the nutritional status of the child. With regard to preschool consultations, the mother was questioned whether she had participated in the various curative and preventive activities for the protection of the health of the child under 5 and the monitoring and the promotion of the child' nutritional status and growth.
2. Dietary practices: age of introduction of complementary diets (considered precocious when this introduction was made before 6 months), age of breastfeeding's cessation (considered precocious when this stop was done before 6 months), and number of daily meals [20]. The assessment of age of introduction of complementary diets was made by asking the mother if at what age the child had received solid, semisolid, or soft foods. Age of breastfeeding's cessation was assessed by asking the mother at what age the child stopped breastfeeding. The evaluation of the minimum number of daily meals was done by asking the mother the minimum number of times the child had received solid, semisolid, or soft foods per day.
3. Mother's sociodemographics characteristics: maternal age, parity, marital status (singleton or union), occupational status (divided into occupied and unoccupied), and level of schooling (was considered low when the mother had reached no more than 6 years of schooling).
4. Father's sociodemographics characteristics: occupation (distributed as occupied and unoccupied) and level of schooling (was considered low when the father had reached no more than 6 years of study). In addition, we looked for parents, that is to say whether one or both biological parents were alive and the children were orphans or not.
5. Family history: family history of malnutrition, number of children under 5 in the family and family size (defined by the number of people in the family and living under the same roof).

### 2.3. Statistical Analyses

The STATA 12 software was used for the various statistical analyses. To determine the predictors of SAM, we performed an univariate analysis using the chi-square test or Fisher's exact test; then, we performed a multivariate analysis.

Variables with a *p* value less than 0.05 in the univariate analysis were included in the logistic regression model using the stepwise method. In the final model, we used variables whose significance level was less than 0.05.

Score's discrimination was assessed using the ROC and C-index curves, and the score was calibrated using the Hosmer–Lemeshow test. We determined the sensitivity, specificity, and percentage of correctly classified cases compared to the C-index. The robustness of the model coefficients was evaluated by bootstrap. The predictive risk score was deduced from the statistical analysis and was established by assigning points to each risk factor retained in the logistic model. To make it simple to use, the score was achieved by using rounded values of these coefficients. The risk probabilities of SAM based on the values of the constructed score were calculated.

### 2.4. Ethics Considerations

The study was authorized by the Medical Ethics Committee of the University of Lubumbashi and the Health authority of Jason Sendwe Hospital before data collection. Patient record/information was anonymized and deidentified prior to analysis to ensure confidentiality of individual patient information.

## 3. Results

Table 1 shows that SAM was more important when the child was born with a low weight (<2500 grams), when the child has a history of repetitive/chronic diarrhea, when the child stopped breastfeeding before 6 months, when the child started complementary feeding before 6 months, when the child receives less than 3 meals a day, when the child does not attend preschool consultations, when the child was an orphan, when the child has a history of family malnutrition, when the number of children under 5 was over 2, when the family size was over 6, when the mother's age was less than 25 years, when the mother's parity was less than 5, when the mother was single, when the parents' level of schooling was low, and when the parents were unemployed.

After logistic regression, nine criteria stand out as predictors of SAM: low birth weight, history of recurrent or chronic diarrhea, daily meal's number less than 3, age of breastfeeding's cessation less than 6 months, age of introduction of complementary diets less than 6 months, maternal age below 25 years, parity less than 5, family history of malnutrition, and number of children under 5 over 2 (Table 2).

Each risk factor was weighted by a regression coefficient representing the weight of the variable in the score calculation. The set of scores obtained is shown in Table 3.

**Figure 1:**
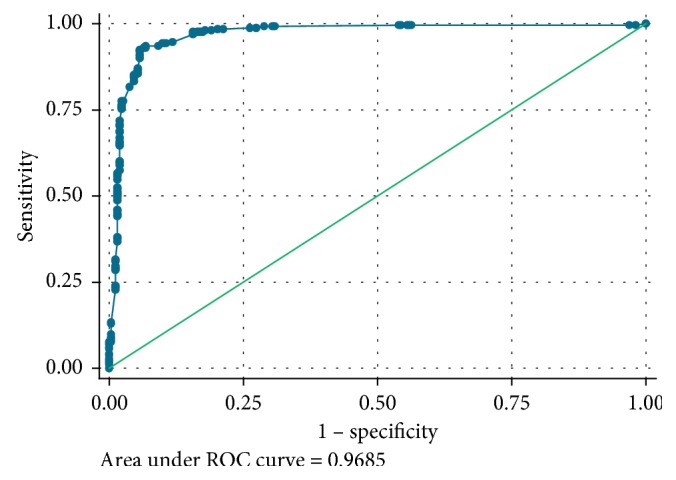
ROC curve showing the performance of SAM predictive score.

The predictive score of SAM was constructed from the logistic model (Table 3). The area under the ROC curve is 0.9685 (Figure 1), which shows exceptional discrimination in terms of its ability to discriminate against children who are going to present SAM to those who are not going to present it.

Presence of these nine criteria affects a certain number of points; the total is 18 points. For each child, the score ranges from 0 to 18 and the higher it is, the higher the risk of SAM.

**Figure 2:**
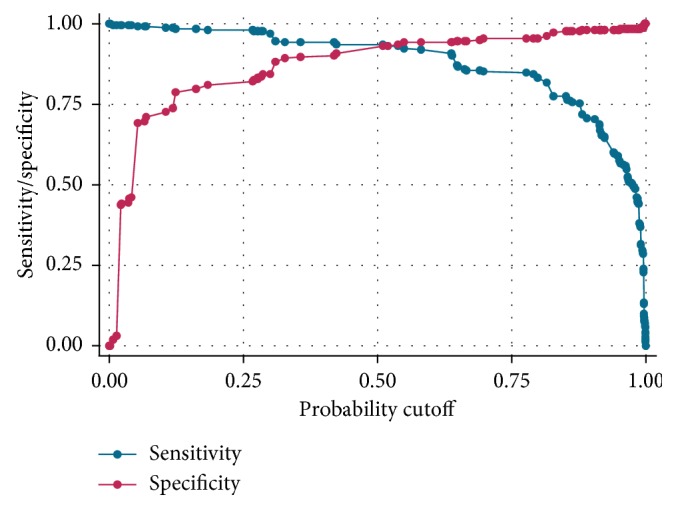
Sensitivity and specificity of SAM predictive score.

Risk probabilities of SAM based on the constructed score values were calculated and are presented in Table 3. A score less than 6 points defines children at low risk of SAM, a score between 6 and 8 points defines a moderate risk of SAM, and a score beyond 8 points presents a high risk of SAM. Thus, a sensitivity of 93.54% was obtained for a specificity of 93.16%, which means that with this threshold, only 6.46% of the children presenting the MAS did not obtain a positive score and 6.84% of children without SAM had a false positive score (Figure 2). The positive predictive value was 93.18%.

## 4. Discussion

In this study, we found multifactorial risk for S in children under 5, which are broadly consistent with those reported in several studies in developing countries. In accordance with other studies [21–26], low birth weight was found to be a factor of SAM. This could be explained by the fact that, often, the low birth weight in our environment results from maternal malnutrition [27], which implies that the conditions in which the child will live are precarious from the point of view of food security but also of feeding and environmental hygiene practices. It is not surprising that a child who is already malnourished before birth and who lives in these conditions will suffer from malnutrition that persists or worsens.

In our series, the history of recurrent or chronic diarrhea was significantly associated with the occurrence of malnutrition, which is similar to the results of several authors [28–30]. This can be explained by the fact that diarrhea is accompanied by a decrease in appetite and a decrease in the absorption of nutrients in the digestive tract, thus achieving a real vicious cycle diarrhea malnutrition and more largely infection malnutrition. Poor nutritional status increases the severity, duration, and incidence of diarrheal episodes.

As noted in previous studies [30–35], our study also showed that early cessation of breastfeeding and early introduction of supplemental feeding were significantly associated with SAM. This early breastfeeding's cessation is often decided abruptly during a child's illness or because of a new pregnancy, thus disrupting the nutritional balance of the child and consequently leading to a state of malnutrition. A study in China showed that introducing other foods before 6 months of age increased the prevalence of diarrheal diseases and pneumonia [36].

We found a significant association between less than three meals a day and SAM. A study conducted in Benin had highlighted the significant association between malnutrition and the quantitative defect in the last 24 hours of food intake [37]. This can be explained by the fact that a good diet must meet some conditions including a good quality, a sufficient quantity, and a frequency of meals acceptable.

In addition, the high number of children under 5 in siblings was found to be a predictor of child malnutrition even after multivariate analyzes. A similar conclusion has been reported in several studies [24, 38, 39]. This association can be explained by the fact that having a large number of young children requires a lot of attention and resources for food and health care. The increase in the number of children in the family is a heavy burden on household resources especially on food and finances reducing as well as the time and quality of care received by children [24, 40].

We found an association between the family history of malnutrition and the occurrence of SAM, which is consistent with the results of studies elsewhere [37, 41]. This can be explained by the fact that a family history of malnutrition can reflect poor living and feeding conditions in this family, thus exposing all other family members to the same risk and especially young children.

We found that the young age of the mother (<25 years old) and low parity (<5) influenced the occurrence of SAM. Ayaya et al. also found that maternal age below 25 years is a risk factor for severe malnutrition [42]. Other authors also point out that maternal age and parity are positive and significant factors in the nutritional status of children and report that children born to young mothers are more likely to suffer health problems than children born to adult women [43]. This association can be explained by the difficulties generally experienced by new mothers (especially young mothers) in taking care of a household, a child, the health of the child, and providing adequate care for their children (often the first child) especially when stopping breastfeeding. Breastfeeding's cessation and introduction of complementary diets are often poorly conducted, leading to deterioration in the nutritional status of children during this period. These mothers have a low level of knowledge of the nutritional needs of the child with regard to the nutritional values of different types of foods given to the child. This information is often given to mothers during preschool consultations. Preschool consultation is the central hub of the monitoring and promotion of children's growth activities from childbirth to the age of 59 months.

We identified different variables that allowed us to establish the predictive risk score for SAM in children under 5. The analysis of the ROC curve led us to define a threshold that is both sensitive and specific enough to detect children at risk of having SAM.

This threshold remains limited to a sensitivity of 93.54% for a specificity of 93.16%. Our score predicts SAM in more than 9 out of 10 malnourished children, but falsely classifies as malnourished 6.84% of well-nourished children. The results of our study come from the analysis of data collected on the population of a single hospital. Nevertheless, the hospital receives sick children referred from almost the entire southeastern part of the DRC. This study should also be conducted in other regions of the DRC and Africa in the short term to evaluate and validate the performance of this model on different populations (transportability). As a result, the model presented here does not claim to have universal validity. Children may be subjected to this tool during vaccination campaigns in the community or during preschool consultations. Those who are at high risk can be followed, and their mothers will receive advice and information on the child's nutritional needs and the nutritional values of the different types of foods given to the child.

The prediction of SAM in at-risk children in developing countries such as ours is very important given all the consequences of SAM on the future of a child. Hence, there is a need for a predictive score to guide screening and the potential decision-making process early before the child is in this pathological state. This means that decisions based on rating score parameters that use easy-to-use variables as proposed in this article can make the difference between life and death for the child. As a result, health workers in low-resource settings where most of the child morbidity and mortality rates are observed are the ideal users for this score. The advantage of the proposed score is that the parameters can be easily recorded during a routine preschool visit or during vaccination campaigns by health workers. This score has the advantage of being rated in hospitals and in nonhospital settings (in the community).

## 5. Conclusion

This multifactorial study of the factors favoring SAM makes it possible to propose a predictive score of onset that is based on covariates that are easy to collect before any hospitalization or even during routine preschool consultations. It is also the first to propose a tool that is important for its use in screening for the risk of SAM before it occurs in our context. No published scores are suitable for predicting the risk of SAM in a population under 5 in developing countries. We therefore propose a simple and effective score, predictive of the risk of SAM that will require an external validation study, that is to say in a population different from the one used to establish it. This score would be a useful and simple clinical tool for targeting the population at risk, limiting the high rates of malnutrition and reducing morbidity and infant mortality in developing countries.

## Figures and Tables

**Table 1:** Bivariate analysis of risk factors for malnutrition among children aged 6–59 months in Lubumbashi (DRC).

Variable	Malnourished children (*n* = 263)	Well-nourished children (*n* = 263)	Crude OR [95% CI]	*p* value
	*n* (%)	*n* (%)		
Child's age <24 months	187 (71.1)	182 (69.2)	1.09 [0.75–1.59]	0.6340
Male sex	161 (61.2)	164 (62.4)	1.04 [0.73–1.49]	0.7879
Low birth weight	124 (47.2)	20 (7.6)	10.83 [6.46–18.16]	<0.000001
History of recurrent/chronic diarrhea	189 (71.9)	28 (10.7)	21.43 [13.32–34.47]	<0.000001
Age of breastfeeding's cessation <6 months	24 (9.1)	3 (1.2)	8.70 [2.58–29.27]	<0.0001
Age of introduction of complementary diet <6 months	234 (89.0)	121 (46.0)	9.46 [6.00–14.93]	<0.000001
Daily meal's number <3	230 (87.5)	58 (22.1)	24.63 [15.44–39.29]	<0.000001
No follow-up of preschool consultations	193 (73.4)	22 (8.4)	30.20 [18.04–50.55]	<0.000001
Orphan	46 (17.5)	10 (3.8)	5.36 [2.64–10.88]	<0.00001
Family history of malnutrition	136 (51.7)	10 (3.8)	27.09 [13.77–53.29]	<0.000001
Over 2 children under 5 in the family	94 (35.7)	7 (2.6)	20.34 [9.21–44.91]	<0.000001
Family size over 6 persons	119 (45.2)	40 (15.2)	4.60 [3.04–6.97]	<0.000001
Mother's age <25 years	81 (30.8)	9 (3.4)	12.56 [6.14–25.66]	<0.000001
Parity <5	117 (44.5)	42 (16.0)	4.21 [2.79–6.35]	<0.000001
Single mother	98 (37.3)	18 (6.8)	8.08 [4.71–13.87]	<0.000001
Unemployed mother	242 (92.0)	124 (47.2)	12.91 [7.77–21.45]	<0.000001
Mother's low level of schooling	174 (66.2)	39 (14.8)	11.22 [7.33–17.18]	<0.000001
Unemployed father	182 (69.2)	140 (53.2)	1.97 [1.38–2.82]	0.0002
Father's low level of schooling	108 (41.1)	10 (3.8)	17.62 [8.94–34.72]	<0.000001

**Table 2:** Logistic regression model of risk of SAM.

Variable	Adjusted OR	95% CI	Coefficient	Score
Low birth weight	2.72	1.18–6.26	1.00	1
History of recurrent/chronic diarrhea	10.34	4.94–21.62	2.33	2
Daily meal's number <3	9.86	4.66–20.85	2.28	2
Age of breastfeeding's cessation <6 months	9.08	1.63–50.62	2.20	2
Age of introduction of complementary diet <6 months	3.19	1.38–7.35	1.16	1
Mother's age <25	16.60	5.92–46.56	2.80	3
Parity <5	6.03	2.27–16.04	1.79	2
Family history of malnutrition	24.89	8.77–70.63	3.21	3
Over 2 children under 5 in the family	5.39	1.66–17.47	1.68	2

**Table 3:** Likelihood of SAM by score according to logistic regression model.

Score	Likelihood of SAM^*∗*^ (%)
0	0.22
1	0.58
2	1.53
3	3.93
4	9.74
5	22.14
6	42.82
7	66.36
8	83.86
9	93.19
10	97.30
11	98.95
12	99.60
13	99.84
14	99.94
15	99.97
16	99.99
17	99.99
18	99.99

^*∗*^Obtained from the formula: *p*=(1/1)+exp(6.1+0.9685 × score).

## Data Availability

The dataset used to support the findings of this study are available from the corresponding author upon request.

## Abbreviations

DRC: Democratic Republic of Congo
ROC: Receiver operating characteristic
SAM: Severe acute malnutrition
SD: Standard deviation
WHO: World Health Organization.
